# Diacerein inhibits the synthesis of resorptive enzymes and reduces osteoclastic differentiation/survival in osteoarthritic subchondral bone: a possible mechanism for a protective effect against subchondral bone remodelling

**DOI:** 10.1186/ar2444

**Published:** 2008-06-25

**Authors:** Christelle Boileau, Steeve Kwan Tat, Jean-Pierre Pelletier, Saranette Cheng, Johanne Martel-Pelletier

**Affiliations:** 1 Osteoarthritis Research Unit, University of Montreal Hospital Centre, Notre-Dame Hospital, 1560 Sherbrooke Street East, Montreal, Quebec, H2L 4M1, Canada

## Abstract

**Introduction:**

Subchondral bone alterations represent an essential component of osteoarthritis (OA). Modifying the abnormal subchondral bone metabolism may be indicated to treat OA. We investigated the effect of diacerein and rhein on the changes occurring in subchondral bone during OA. To this end, we determined the drugs' effects on metalloprotease-13 (MMP-13) synthesis on subchondral bone and on the osteoblast signalling pathways. In osteoclasts, we studied MMP-13 and cathepsin K production as well as cell differentiation, proliferation, and survival.

**Methods:**

The effect of diacerein/rhein on the production of subchondral bone MMP-13 was determined by enzyme-linked immunosorbent assay. Signalling pathways were evaluated on osteoblasts by Western blot. Osteoclast experiments were performed using cells from the pre-osteoclastic murine cell line Raw 264.7. Osteoclast MMP-13 and cathepsin K activities were determined by specific bioassays and differentiation of these cells quantified by tartrate-resistant acid phosphatase staining.

**Results:**

Diacerein and rhein reduced, in a dose-dependent manner, the interleukin-1-beta (IL-1β)-induced MMP-13 production in OA subchondral bone. This effect occurred through the inhibition of ERK1/2 (extracellular signal-regulated kinase-1/2) and p38. In osteoclasts, they significantly reduced the activity of MMP-13 and cathepsin K. Moreover, these drugs effectively blocked the IL-1β effect on the osteoclast differentiation process and the survival of mature osteoclasts.

**Conclusion:**

Altogether, these data suggest that diacerein/rhein could impact the abnormal subchondral bone metabolism in OA by reducing the synthesis of resorptive factors and osteoclast formation.

## Introduction

Osteoarthritis (OA) is considered a complex illness. Although we may not yet completely know all of the initiating factors involved in the degeneration of the articular tissues, significant progress regarding the etiopathogenesis of this disease has been made. For decades, the prevailing concept has centered on the destruction of the articular cartilage. There is now substantial evidence not merely that alterations in the subchondral bone metabolism are secondary manifestations of OA, but that they comprise an integral component of the disease, and data suggest a key role played by the subchondral bone in the initiation and/or progression of articular tissue degeneration.

Several reports have indicated that the subchondral bone remodelling that occurs during OA involves both bone resorption and bone formation. Studies allowing chronological evaluation in animal models have suggested a predominance of bone formation in the more advanced stage of the disease [1-4], while, in contrast, the remodelling in the early phase favors bone resorption [4-6]. This latter finding agrees with the study of Bettica and colleagues [7], who demonstrated that, *in vivo *in humans, general bone resorption is increased in patients with progressive knee OA. Similarly, Messent and colleagues [8], with the use of fractal signature analysis, showed that bone loss occurred in patients with knee OA and that changes were associated with an increase in the number and size of the remodelling units.

*In vitro *studies have also demonstrated that the subchondral bone is the site of several dynamic morphological changes that appear to be part of the OA process. These changes are allied with many local abnormal biochemical pathways, including the increased synthesis of several bone markers, growth factors, cytokines, proteases, and inflammatory mediators. The levels of alkaline phosphatase, osteocalcin, type I collagen, interleukin (IL)-6, transforming growth factor-beta, prostaglandin E_2 _(PGE_2_), leukotriene B_4_, and proteases, including urokinase, cathepsin K, and the metalloprotease (MMP)-13, have all been found to be elevated in human OA subchondral bone osteoblasts [6,9-13].

The pharmacological treatments for OA are centered mainly on the use of analgesics and non-steroidal anti-inflammatory drugs (NSAIDs). These are symptomatic agents that, thus far, have been shown to be solely capable of relieving the signs and symptoms of the disease. Due primarily to recent progress in understanding the disease, new approaches for the treatment of OA are now being explored. Compounds that inhibit one or more OA disease processes are under evaluation for their potential to alter the degenerative changes. As the subchondral bone alterations also appear to contribute to cartilage deterioration [14], therapeutic strategies aimed at modifying the abnormal metabolism of the subchondral bone cells may have significant impact on the treatment of OA.

Diacerein, a drug of the anthraquinone class, has rhein as its active metabolite. In chondrocytes, this drug acts on the IL-1β system, reducing the level of this cytokine as well as downregulating the IL-1β-induced inflammatory pathways and cartilage breakdown in OA [15-18]. On human subchondral bone osteoblasts, data showed that diacerein/rhein reduces osteocalcin, urokinase, and IL-6, factors that would contribute to curbing bone formation/resorption [19].

This study aims at providing a more complete and comprehensive understanding of the effects of diacerein/rhein on OA subchondral bone metabolism and cells (osteoblasts and osteoclasts). As bone resorption is mediated by several processes, including the synthesis of proteases that can induce matrix degradation and osteoclast differentiation and proliferation, our study aimed first to investigate the effects of diacerein/rhein on the synthesis of major proteases involved in bone remodelling/resorption, namely MMP-13 and cathepsin K. Moreover, we sought to gain new insight into the effects of the drug on the bone resorptive process.

## Materials and methods

### Specimen selection

Subchondral bone was obtained from OA patients who had undergone total knee replacement surgery. Specimens were taken from weight-bearing areas of the femoral condyles. Subchondral bone specimens were dissected away from the remaining cartilage and trabecular bone under sterile conditions as previously described [9,10]. A total of 16 patients (72 ± 9 years old, mean ± standard deviation; 6 males and 10 females) classified as having OA according to recognized American College of Rheumatology clinical criteria were included in this study [20]. At the time of surgery, the patients had symptomatic disease requiring medical treatment in the form of acetaminophen, NSAIDs, or selective cyclooxygenase-2 inhibitors. None had received intra-articular steroid injections within 3 months prior to surgery, and none had received medication that would interfere with bone metabolism. The institutional Ethics Committee Board of the University of Montreal Hospital Centre approved the use of the human articular tissues.

### Subchondral bone tissue explant

#### Culture conditions

Subchondral bone explants of about 5 × 3 mm were placed in 24-well plates containing BGJb medium (Invitrogen Life Technologies, Burlington, ON, Canada) supplemented with 2% fetal bovine serum (FBS) (Invitrogen Life Technologies) and an antibiotic mixture (100 units per milliliter penicillin base and 100 μg/mL streptomycin base) (Multicell; Wisent, St-Bruno, QC, Canada). The explants were treated with or without IL-1β (5 ng/mL) and therapeutic concentrations of diacerein (10 or 20 μg/mL) or rhein (10 or 20 μg/mL) for 5 days at 37°C in a humidified atmosphere of 5% CO_2_/95% air. At the end of the incubation period, culture medium was collected and MMP-13 levels were determined using a specific enzyme-linked immunosorbent assay (ELISA). The MMP-13 ELISA was from Amersham Biosciences (now part of GE Healthcare Bio-Sciences Inc., Baie-d'Urfé, QC, Canada) and recognized both the pro and active forms of the enzyme, the sensitivity being 32 pg/mL. The level was expressed as a fold expression compared with the IL-1β, which was assigned a value of 1.

#### Immunostaining

Subchondral bone explants were fixed as previously described [6] in Tissufix #2 (Chaptec, Montreal, QC, Canada), decalcified in Rapid Bone Decalcifier RDO (Apex Engineering Products Corporation, Aurora, IL, USA), and embedded in paraffin. Sections (5 μm) were placed on Superfrost Plus slides (Fisher Scientific, Nepean, ON, Canada). Slides were deparaffinized in toluene, rehydrated in a reverse-graded series of ethanol, and pre-incubated with chondroitinase ABC (0.25 U/mL; Sigma-Aldrich, St. Louis, MO, USA) in phosphate-buffered saline (PBS) (pH 8.0) for 60 minutes at 37°C. Subsequently, the specimens were washed in PBS, placed in 0.3% TritonX-100 in PBS for 20 minutes and in 3% hydrogen peroxide/PBS for another 15 minutes. Slides were further incubated with a blocking serum (Vectastain ABC kit; Vector Laboratories Inc., Burlingame, CA, USA) for 60 minutes, blotted, and then overlaid with the primary antibody against MMP-13 (15 μg/mL; R&D Systems, Minneapolis, MN, USA) or cathepsin K (1 μg/mL; Novocastra, now part of Leica Microsystems, Wetzlar, Germany) for 18 hours at 4°C in a humidified chamber. Each slide was washed three times in PBS (pH 7.4) and stained using the avidin-biotin complex method (Vectastain ABC kit). The color was developed with 3, 3'-diaminobenzidine (DAB) (DAKO Diagnostics Canada Inc., Mississauga, ON, Canada) containing hydroxide peroxide. Slides were counterstained with hematoxylin/eosin.

The staining specificity of the antibody used was determined using three controls according to the same experimental protocol: (a) use of absorbed immune serum (1 hour at 37°C) with a 20-fold molar excess of human recombinant MMP-13 (R&D Systems) or cathepsin K (Calbiochem, now part of EMD Biosciences, Inc., San Diego, CA, USA), (b) omission of the primary antibody, and (c) substitution of the primary antibody with an autologous pre-immune serum. These controls showed only background staining.

### Subchondral bone osteoblasts

#### Culture

Subchondral bone osteoblasts were prepared as previously described following a collagenase digestion procedure [9,10]. Briefly, subchondral bone specimens were digested by sequential collagenase type I digestion, followed by cell culture in BGJb medium containing 20% FBS. At confluence, primary osteoblasts were split once into 24-well plates at a final cell density of 50,000 cells per square centimeter. Cells were fed with BGJb medium, supplemented with an antibiotic mixture (100 U/mL penicillin and 100 μL/mL streptomycin; Multicell) and 10% FBS until confluence. Only first passaged cells were employed.

Signalling pathway experiments were conducted on osteoblasts pre-treated by therapeutic concentrations of diacerein or rhein at 20 μg/mL for 2 hours and treated with IL-1β (100 pg/mL) for an additional 30 minutes. The levels of the phosphorylated mitogen-activated protein (MAP) kinases, extracellular signal-regulated kinase-1/2 (ERK1/2), p38, and stress-activated protein kinase/c-jun N-terminal kinase (SAPK/JNK) (p46 and p54) were determined on the cell lysate by Western blot as described below.

#### Western blotting

Total proteins were extracted with 0.5% SDS (Invitrogen Life Technologies) supplemented with protease inhibitors. The protein level was determined using the bicinchoninic acid protein assay, and 10 μg of the protein was electrophoresed on a 12% SDS gel polyacrylamide. The proteins were transferred electrophoretically onto a nitrocellulose membrane (Bio-Rad Laboratories [Canada] Ltd., Mississauga, ON, Canada) for 1 hour at 4°C. The efficiency of transfer was controlled by a brief staining of the membrane with Ponceau red and destained in water and TTBS 1× (Tris 20 mM, NaCl 150 mM, pH 7.5, and 0.1% Tween 20) before immunoblotting.

The membranes were incubated overnight at 4°C with 5% skimmed milk in SuperBlock^® ^Blocking Buffer in Tris-Buffered Saline (Pierce, Rockford, IL, USA) or in TTBS 1× only. The membranes were then washed once with TTBS 1× for 10 minutes and incubated in SuperBlock^® ^Blocking Buffer and TTBS 1× (Superblock^® ^1:10 with TTBS 1×) with a mouse anti-phospho ERK1/2 (dilution: 1:2,000; Thr 202/Tyr 204; Cell Signaling Technology, Inc., Danvers, MA, USA), a rabbit polyclonal anti-phospho p38 (dilution: 1:500; Thr 180/Thr 182; Biosource, Nivelles, Belgium), and a mouse anti-phospho SAPK/JNK (dilution: 1:1,000; Thr 183/Tyr 185; New England Biolabs Ltd., Pickering, ON, Canada) overnight at 4°C. The membranes were washed with TTBS 1× and incubated for 1 hour at room temperature with the second antibody. The secondary antibodies were anti-mouse or anti-rabbit IgG (dilution: 1:50,000; Pierce). They were then washed again with TTBS 1×. Detection was performed by chemiluminescence using the Super Signal^® ^ULTRA chemiluminescent substrate (Pierce) and exposure to Kodak Biomax photographic film (GE Healthcare Bio-Sciences Inc.). The band intensity was measured by densitometry using TotalLab TL100 Software (Nonlinear Dynamics Ltd, Newcastle upon Tyne, UK), and data are expressed as fold difference with respect to the IL-1β control, which was assigned a value of 1.

### Osteoclasts from Raw 264.7 cells

#### Culture conditions

Raw 264.7 cells (American Type Culture Collection, Manassas, VA, USA) were seeded at a density of 10,000 cells per well in 24-well plates with Dulbecco's modified Eagle's medium culture medium (Multicell) supplemented with the antibiotic mixture, 10% FBS, and 1% sodium pyruvate (Multicell). Cells were treated with receptor activator of nuclear factor-κB ligand (RANKL) (100 ng/mL; R&D Systems) from the first day of culture and for the entire duration of the experiment. RANKL allows the pre-osteoclast Raw 264.7 cells to differentiate into mature osteoclasts after 5 days of culture. The culture medium was changed every 2 days.

On the fifth day, RANKL-treated cells were co-incubated with or without IL-1β (100 pg/mL) and therapeutic concentrations of diacerein or rhein at 10 or 20 μg/mL for 2 days at 37°C in a humidified atmosphere of 5% CO_2_/95% air. At the end of the incubation period, the conditioned medium served for MMP-13 determination, and cathepsin K determination was carried out on the cell lysates. Quantification of mature osteoclasts was also performed on other cell cultures under the same experimental conditions. At the end of the incubation period, the osteoclasts were fixed with citrate/acetone solution and stained for tartrate-resistant acid phosphatase (TRAP) according to the manufacturer's recommendation (Sigma-Aldrich). Osteoclast formation was quantified by counting, under a light microscope, the newly differentiated multinucleated TRAP-positive cells containing at least three nuclei.

#### Determination of functional metalloprotease-13 and cathepsin K

Since MMP-13 and cathepsin K are produced by the murine cell line Raw 264.7, proteins not recognized by the commercially available ELISAs, determinations were performed using activity assays specific for each protease. Functional MMP-13 levels were determined using the MMP-13 activity assay (Chemicon International, Temecula, CA, USA) according to the manufacturer's instructions. MMP-13 was activated by APMA (p-aminophenyl mercuric acetate) (0.5 mM) at 37°C for 60 minutes prior to the assay. For cathepsin K, the cell lysates were harvested in the specific assay buffer according to the manufacturer's instructions and the levels were determined using the Bioassay™ assay (United States Biological Inc., Swampscott, MA, USA). Data are expressed as fold difference with respect to the IL-1β, which was assigned a value of 1.

#### Mature osteoclast survival

Raw 264.7 cells were treated with RANKL (100 ng/mL) from the first day of culture and for the entire duration of the experiment. The culture medium was changed every 2 days. On the fifth day, RANKL-treated cells were incubated with or without IL-1β (100 pg/mL) and therapeutic concentrations of diacerein or rhein at 20 μg/mL for 2 days. At the end of the incubation period, the osteoclasts were fixed and the number of TRAP-positive cells was determined as described above. Results were calculated as the number of differentiated osteoclasts per well.

#### Osteoclast differentiation and proliferation

Raw 264.7 cells were treated with RANKL (100 ng/mL) as well as with IL-1β (100 pg/mL) and therapeutic concentrations of diacerein or rhein at 20 μg/mL from the first day of culture and for the entire duration of the experiment. The culture medium was changed every 2 days. On the seventh day, the osteoclasts were fixed and the number of TRAP-positive multinucleated cells was determined as described above.

### Statistical analysis

Results were expressed as the mean ± standard error of the mean of independent specimens, and assays were performed in duplicate. Statistical analysis was performed using the two-tailed paired Student *t *test, and a difference of less than or equal to 0.05 was considered significant.

## Results

### Subchondral bone immunostaining

**Figure 1:**
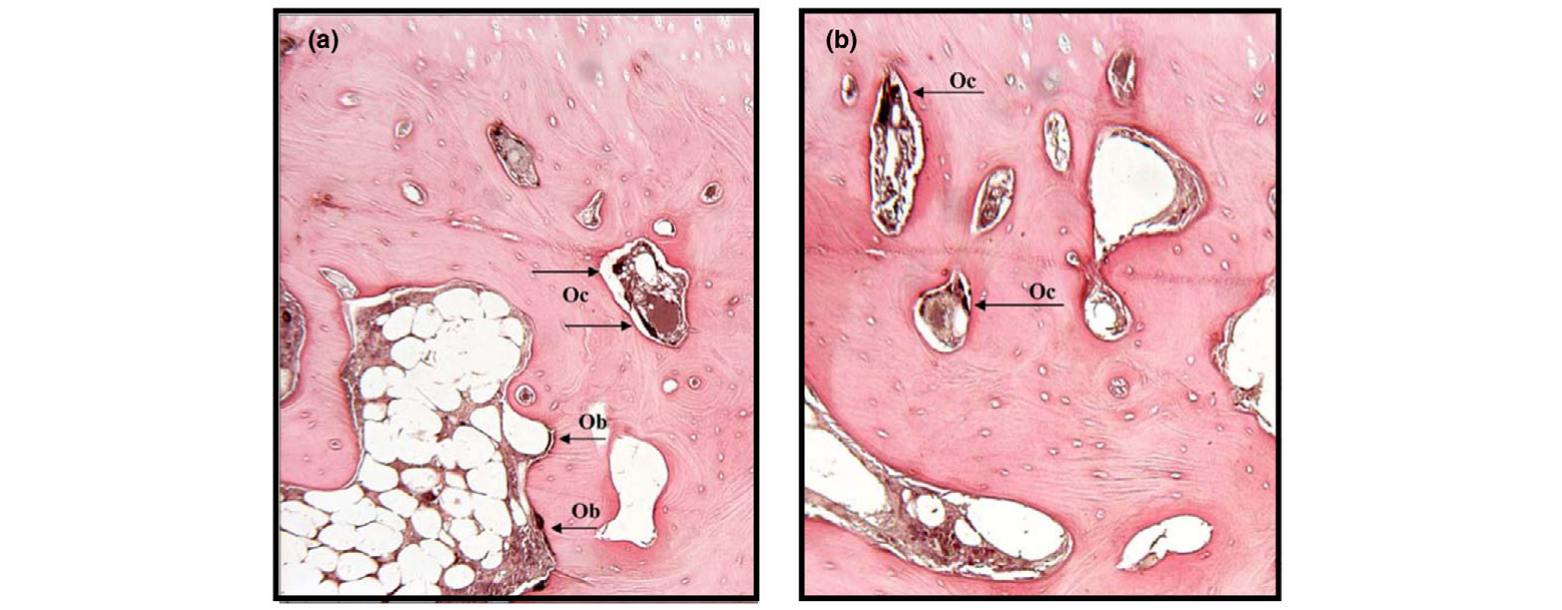
Representative immunohistochemical staining section for **(a) **metalloprotease-13 (MMP-13) and **(b) **cathepsin K in human osteoarthritis subchondral bone. MMP-13 was detected in the osteoblasts (Ob) as well as in the osteoclasts (Oc). Cathepsin K was detected only in osteoclasts. Original magnification, ×100.

To verify the production of MMP-13 and cathepsin K in human OA subchondral bone, immunostaining for each of these two proteases was performed. Data (n = 3) revealed that both proteases are produced and that MMP-13 was detected in both osteoblasts and osteoclasts, whereas cathepsin K was detected only in osteoclasts (Figure 1).

### Effect of diacerein/rhein on metalloprotease-13 synthesis in osteoarthritis subchondral bone

**Figure 2:**
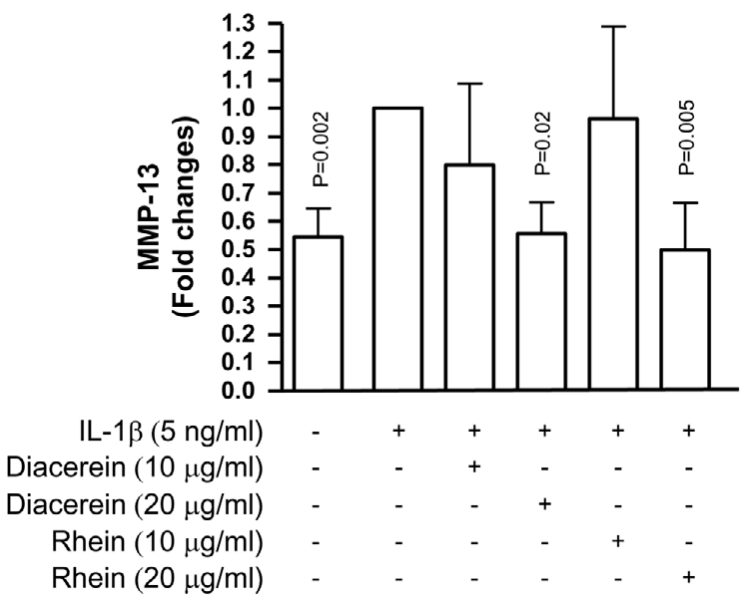
Effect of diacerein and rhein on metalloprotease-13 (MMP-13) production in human osteoarthritis subchondral bone. Subchondral bone explants were incubated for 5 days with or without interleukin-1-beta (IL-1β) (5 ng/mL) and diacerein or rhein (10 or 20 μg/mL). Data are expressed as fold changes compared with IL-1β-treated control, which was assigned a value of 1. Statistical analysis was performed versus IL-1β-treated control.

As illustrated in Figure 2, the synthesis of MMP-13 in subchondral bone explants (n = 5 to 9) was significantly upregulated by IL-1β. Diacerein and rhein reduced, in a dose-dependent manner, the production of the IL-1β-induced MMP-13. The effect reached statistical significance with the highest tested dose (20 μg/mL).

### Effect of diacerein/rhein on intracellular signalling pathways

**Figure 3:**
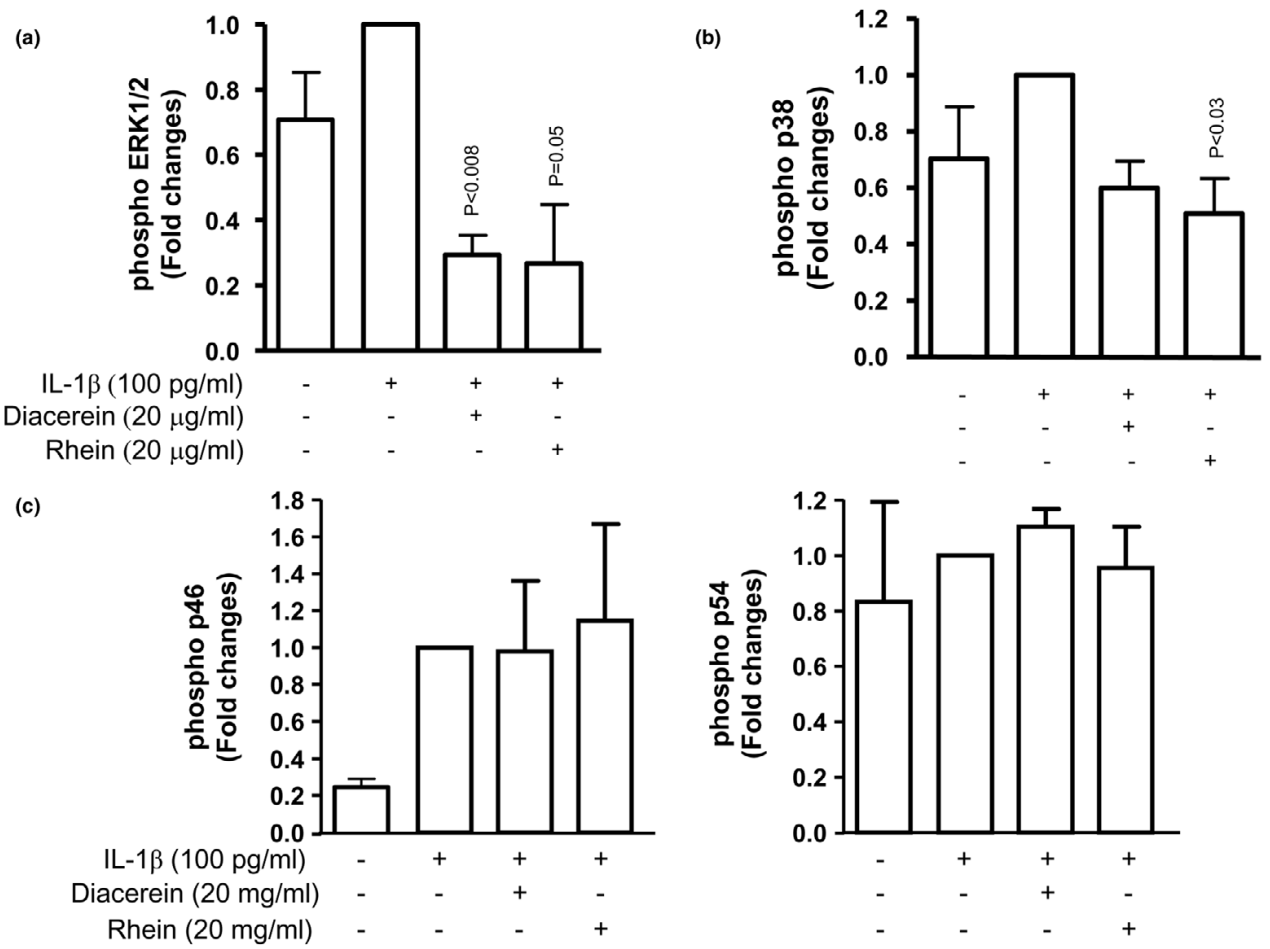
Effect of diacerein and rhein on subchondral bone osteoblast intracellular mitogen-activated protein (MAP) kinase pathways. Subchondral bone osteoblasts were pre-incubated for 2 hours with diacerein or rhein at 20 μg/mL and incubated for 30 minutes in the presence or absence of interleukin-1-beta (IL-1β) (100 pg/mL). Levels of phosphorylated **(a) **extracellular signal-regulated kinase-1/2 (ERK1/2), **(b) **p38, and **(c) **stress-activated protein kinase/c-jun N-terminal kinase (SAPK/JNK) (p46 and p54) MAP kinases were studied by Western blot and quantified by densitometry as described in Materials and methods. Data are expressed as fold changes compared with IL-1β-treated control, which was assigned a value of 1. Statistical analysis was performed versus IL-1β-treated control.

To gain insight into the mechanisms of these drugs on the OA subchondral bone osteoblasts, we further studied the effect of the therapeutic concentration of these drugs, 20 μg/mL, on the major intracellular signalling pathways pertinent to OA pathology. On OA subchondral bone osteoblasts, data (n = 3 to 4) showed that, while IL-1β activated the ERK1/2 and p38 pathways (Figures 3a and 3b, respectively), diacerein and rhein both significantly inhibited the phosphorylation levels of ERK1/2 (Figure 3a) and both decreased the p38 phosphorylation with statistical significance reached for rhein. IL-1β also markedly increased the SAPK/JNK (p46 and p54), particularly the level of the p46 isoforms. Diacerein and rhein, however, had no effect on the activation level of either kinase.

### Effect of diacerein and rhein on metalloprotease-13 and cathepsin K in osteoclasts

**Figure 4:**
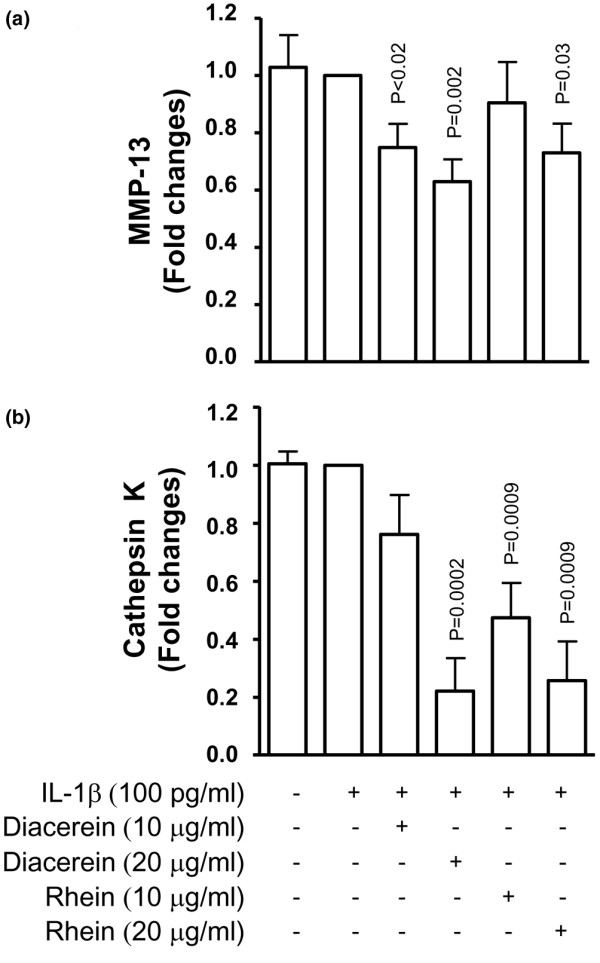
Effect of diacerein and rhein on the osteoclastic levels of **(a) **metalloprotease-13 (MMP-13) and **(b) **cathepsin K. Determination was performed in the conditioned medium for MMP-13 and on cell lysates for cathepsin K. Raw 264.7 cells were incubated for 5 days with RANKL (100 ng/mL), allowing the cells to differentiate into osteoclasts. After this period, the cells were incubated for 2 days together with RANKL in the presence or absence of interleukin-1-beta (IL-1β) (100 pg/mL) and diacerein or rhein (10 or 20 μg/mL). Data are expressed as fold changes compared with IL-1β-treated control, which was assigned a value of 1. Statistical analysis was performed versus IL-1β-treated control. RANKL, receptor activator of nuclear factor-κB ligand.

To better document and discriminate the effect of diacerein and rhein on the different bone cell populations, further experiments were performed on osteoclasts. To this end, a pre-osteoclastic murine cell line, Raw 264.7, was used. These cells, upon stimulation by RANKL, differentiate into multinucleated TRAP-positive osteoclasts [21,22]. As illustrated in Figure 4, stimulation with IL-1β had no effect on the level of MMP-13 produced by Raw 264.7 cells (n = 8). Diacerein and rhein at both concentrations (10 and 20 μg/mL) significantly inhibited the MMP-13 level (Figure 4a). The intracellular level of cathepsin K was not stimulated by IL-1β (n = 8) (Figure 4b). Data showed that both diacerein and rhein significantly decreased the protease activity level in a dose-dependent manner.

### Effect of diacerein and rhein on osteoclast differentiation

#### Survival of differentiated osteoclasts

**Figure 5:**
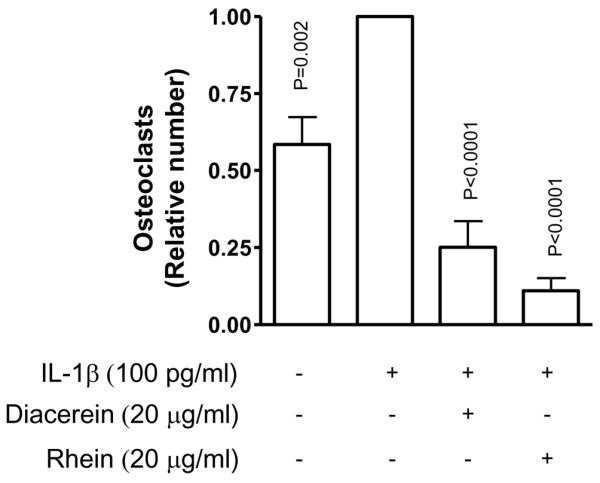
Effect of diacerein and rhein on osteoclast survival. Raw 264.7 cells were incubated for 5 days with RANKL (100 ng/mL) and for an additional 2 days together with RANKL in the presence or absence of interleukin-1-beta (IL-1β) (100 pg/mL) and diacerein or rhein (20 μg/mL). The number of differentiated osteoclasts was determined by the tartrate-resistant acid phosphatase staining assay. Data are expressed as fold changes compared with IL-1β-treated control, which was assigned a value of 1. Statistical analysis was performed versus IL-1β-treated control. RANKL, receptor activator of nuclear factor-κB ligand.

Cells were treated for 5 days with RANKL and then incubated for 2 days together with RANKL in the absence or presence of IL-1β and diacerein or rhein at 20 μg/mL (n = 8). At the end of the incubation period, TRAP staining was performed and the number of TRAP-positive and multinuclear cells was quantified. Data showed (Figure 5) that stimulation with IL-1β significantly increased the number of multinucleated differentiated osteoclasts. Treatment with diacerein or rhein significantly inhibited the IL-1β effect.

#### Differentiation/proliferation of osteoclasts

**Figure 6:**
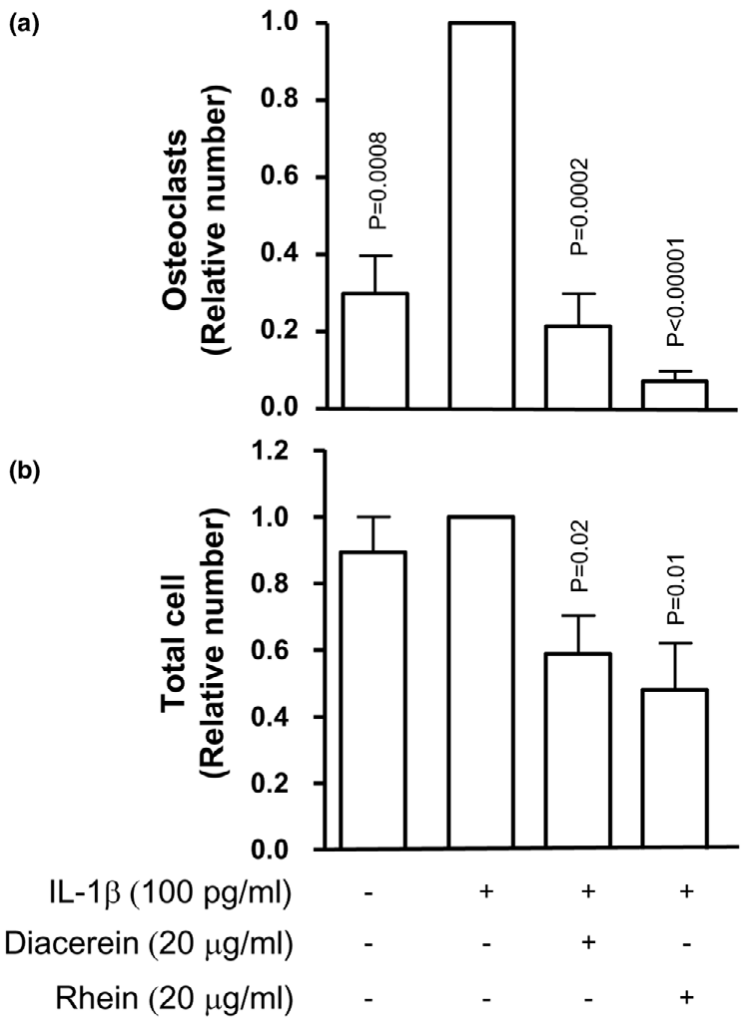
Effect of diacerein and rhein on osteoclast **(a) **proliferation/differentiation and **(b) **total cells. Raw 264.7 cells were incubated for 7 days with RANKL (100 ng/mL) in the presence or absence of interleukin-1-beta (IL-1β) (100 pg/mL) and diacerein or rhein (20 μg/mL). The number of differentiated osteoclasts was determined by the tartrate-resistant acid phosphatase staining assay. Data are expressed as fold changes compared with IL-1β-treated control, which was assigned a value of 1. Statistical analysis was performed versus IL-1β-treated control. RANKL, receptor activator of nuclear factor-κB ligand.

Cells were treated from the first day (before formation of differentiated/mature osteoclasts) with RANKL in the absence or presence of IL-1β and diacerein or rhein at 20 μg/mL (n = 6). After the seventh day of incubation, cells were processed for TRAP staining and multinuclear cells as well as the total number of cells were quantified. As expected, there was a differentiation process of Raw 264.7 cells under RANKL treatment, which was associated with an increase in the rate of osteoclast formation under IL-1β stimulation. Interestingly, diacerein and rhein markedly and significantly inhibited osteoclast differentiation to a level that was even lower than the basal level. Moreover, the drugs also significantly decreased the proliferation rate of the Raw 264.7 cells (Figure 6b) as the total cell number, after 7 days of culture, was significantly lower under treatment with both diacerein and rhein.

## Discussion

Diacerein and rhein have demonstrated positive effects on the IL-1β system in cartilage, and recently a role in bone tissue was suggested [19,23,24]. Based on the findings that joints affected by OA demonstrate an increased bone remodelling process, therapeutic strategies aimed at modifying the abnormal metabolism of bone cells may be indicated for OA. We therefore explored the effects of diacerein and rhein on OA subchondral bone and osteoclasts to determine whether these drugs could alter the abnormal bone remodelling process in this tissue.

In bone, osteoblasts and osteoclasts contribute either alone or in combination to the remodelling process, and the disturbance between the activities of these two cells is suggested to be responsible for the development of an altered bone metabolism. Such disturbance could be due to an upregulation of the proteases, including MMP-13 and cathepsin K, which are potent bone resorptive factors [25-30]. In an OA dog model, the modulation of these proteases was shown to be linked to subchondral bone structural changes [6]. In humans, findings from the present study showed that cathepsin K was present quite selectively in subchondral bone osteoclasts, whereas MMP-13 was detected in the subchondral bone osteoblasts as well as in osteoclasts. These findings concur with the *in situ *localization of these proteases in an OA dog model [6]. Moreover, as MMP-13 is known to work in conjunction with cathepsin K in the induction of bone resorption, their combined effect is likely to be very potent in inducing resorption in the subchondral bone.

IL-1β, a pleiotropic cytokine highly involved during the OA process, is well known to induce the expression of a large variety of pro-inflammatory molecules and cytokines as well as several MMPs, including MMP-13 [26,29,31]. Our data showed that diacerein and its active metabolite, rhein, both inhibited the IL-1β-induced MMP-13 production in human OA subchondral bone. In the same line of thought, a study performed by Legendre and colleagues [32] recently demonstrated a similar inhibitory effect of rhein on MMP-13 production in articular chondrocytes. Hence, findings from these studies support the beneficial effect of rhein on both the subchondral bone and the cartilage.

The mechanisms by which these drugs exert their effect occur through the downregulation of ERK1/2 and p38 MAP kinase activation, but not that of SAPK/JNK. These findings also agree with studies on other cell types demonstrating the critical role of ERK1/2 and p38 activation in the regulation of MMP-13 as well as with data showing that rhein reduces the IL-1β-induced ERK1/2 pathway in bovine chondrocytes [32,33].

Subchondral bone immunohistochemical analysis showed that both MMP-13 and cathepsin K were detected in mature multinucleated osteoclasts. The role of MMP-13 in bone biology is of major importance as, on the one hand, MMP-13 secretion from the osteoblasts could be responsible for increasing type I collagen degradation and, on the other hand, in osteoclasts it could contribute to an increased bone resorption process. Thus, in this tissue, diacerein and rhein could act at two different levels, by limiting the extent of the type I collagen degradation as well as the resorptive activity of the subchondral bone. The role of cathepsin K in the remodelling of this tissue has been well documented and a recent study carried out in a dog OA model [6] reported that this enzyme was not only involved in the subchondral bone but also likely responsible for the resorption of the calcified cartilage. Thus, in osteoclasts, the reduction in activity of these enzymes by the drugs will impact the balance between bone resorption and formation. Interestingly, our data showed that IL-1β on the mature osteoclasts was without effect on the activity of either MMP-13 or cathepsin K, but both drugs significantly decreased their levels. Hence, the exact mechanism by which these drugs act on these proteases in the osteoclasts needs further investigation.

In the context of the remodelling process, we then looked at possible effects of these drugs on osteoclast differentiation and survival processes. Our data showed that, indeed, these drugs have a major role in controlling osteoclastogenesis. This latter process is tightly controlled by some members of the TNF superfamily [34]. In this particular system, RANKL, which is synthesized by the osteoblastic lineage cells, is essential for mediating bone resorption through the enhancement of osteoclast differentiation and proliferation. RANKL stimulates osteoclastogenesis and osteoclast function by binding to the cell surface RANK located on osteoclast precursors and osteoclasts – the interaction necessary for the formation of osteoclasts, osteoclast survival, and bone resorption [35-37].

For our study, a murine cell line, Raw 264.7, was used to investigate osteoclast formation and survival capacity under diacerein/rhein treatment. These cells were chosen as they are in a pre-osteoclast state and do not require any support (for example, dentin) for osteoclast differentiation/formation, but only RANKL treatment [21,22]. For the osteoclast survival capacity, cells were pre-treated for 5 days with RANKL and then the mature osteoclasts were treated with IL-1β. Data showed, as expected, that the number of multinucleated TRAP-positive osteoclasts was highly increased [38-41] and that both drugs negatively modulated the survival capacity of the mature osteoclasts. Diacerein reversed the IL-1β-increased osteoclastogenesis, and rhein further decreased the osteoclast survival below the basal level. The effect of rhein on the basal level could be related to its activity on the apoptotic mechanism of these cells and/or on the cells' membrane functions. Hence, since mature osteoclasts are non-dividing cells, the setup of an apoptotic mechanism is the only final end stage of the differentiated osteoclasts. In this particular cellular and molecular mechanism, caspase-3 has been shown to be involved [42,43]. Therefore, treatment with rhein and/or diacerein could disturb the equilibrium by inducing pro-apoptotic signals as well as caspase-3 activation, thereby accelerating the subsequent apoptotic pathway occurring in the mature osteoclast cells. Indeed, rhein has been found, in certain cancer cells, to induce apoptosis through the activation of caspase-3 [44-46] and also to interact with the cell membrane, resulting in an alteration of membrane-associated functions [47,48].

Further findings showed that diacerein and rhein effectively block not only the survival of mature osteoclasts but also the differentiation and the proliferation processes of pre-osteoclasts into mature osteoclasts. In the presence of IL-1β, which is a potent stimulator of osteoclastic bone resorption [38-41], osteoclast differentiation was greatly induced. Treatment with both diacerein and rhein significantly inhibited the IL-1β effect, and rhein further reduced this differentiation below the basal value. Complementary experiments (data not shown) revealed that these drugs, in the presence of RANKL but without IL-1β, also markedly decreased the differentiation process. These effects could be related to a reduced proliferation rate as the total cell number was significantly less under treatment with diacerein and rhein than the control cells.

Although further studies are needed to fully elucidate the precise mechanism of action of diacerein/rhein on osteoclasts, it could be related to their effect on PGE_2_, the levels of which were shown to be increased by these drugs in many cell types [16,49], including human subchondral bone osteoblasts [19]. Indeed, a previous study reported that high PGE_2 _levels inhibited bone resorption [50] and that human subchondral bone osteoblasts expressing low levels of PGE_2 _enhanced the formation of osteoclasts from the Raw 264.7 cells, whereas those expressing higher levels of PGE_2 _did not. Although such inhibition of high levels of PGE_2 _on osteoclast formation could take place indirectly, it could also act directly on the osteoclast precursors. Indeed, Take and colleagues [51] recently demonstrated the presence of a direct PGE_2_-induced inhibition of osteoclast precursor formation, which occurs through the interaction of PGE_2 _with its specific receptors.

## Conclusion

This study provides evidence that diacerein/rhein treatment could impact the abnormal metabolism in OA subchondral bone by reducing the altered resorptive activity in this tissue. This study brings to light some new and interesting information about the mechanisms by which diacerein/rhein could exert protective effects on OA articular structural changes. However, these *in vitro *findings should be confirmed *in vivo*.

## Abbreviations

ELISA = enzyme-linked immunosorbent assay; ERK1/2 = extracellular signal-regulated kinase-1/2; FBS = fetal bovine serum; IL = interleukin; JNK = c-jun N-terminal kinase; MAP = mitogen-activated protein; MMP = metalloprotease; NSAID = non-steroidal anti-inflammatory drug; OA = osteoarthritis; PBS = phosphate-buffered saline; PGE_2 _= prostaglandin E_2_; RANKL = receptor activator of nuclear factor-κB ligand; SAPK = stress-activated protein kinase; TRAP = tartrate-resistant acid phosphatase; TTBS = Tris 20 mM, NaCl 150 mM, pH 7.5, and 0.1% Tween 20.

## Competing interests

This study was supported by a grant from TRB Chemedica International S.A. (Geneva, Switzerland). J-PP and JM-P have received fees for their consultancy and lecturer services from TRB Chemedica International S.A. The other authors declare that they have no competing interests.

## Authors' contributions

CB participated in study design, acquisition of data, analysis and interpretation of data, manuscript preparation, and statistical analysis. J-PP participated in study design, analysis and interpretation of data, and manuscript preparation. JM-P participated in study design, analysis and interpretation of data, manuscript preparation, and statistical analysis. SKT participated in acquisition of data, analysis and interpretation of data, and manuscript preparation. SC participated in acquisition of data and manuscript preparation. All authors read and approved the final manuscript.
